# Case Report: A “senior” with serpentine-like syndrome—treatment of combined brachyoesophagus, intrathoracic stomach and cervical rachischisis

**DOI:** 10.3389/fped.2024.1378234

**Published:** 2024-09-10

**Authors:** Judith Lohmann, Anna Freund, Tobias Klein, Daniel Acero, Thomas Boemers

**Affiliations:** ^1^ Department of Pediatric Surgery & Pediatric Urology, Children’s Hospital Amsterdamer Straße, Cologne, Germany; ^2^ Pediatric and Neonatal Intensive Care Unit, Children’s Hospital Amsterdamer Straße, Cologne, Germany

**Keywords:** congenital diaphragmatic hernia, hiatal hernia, diaphragmatic defect, vertebral anomaly, enterothorax, asplenia, serpentine-like syndrome, spinal dysraphia

## Abstract

Serpentine-like syndrome, characterized by the combination of intrathoracic stomach, a notably short esophagus anomaly, splenic abnormalities, and cervical spine malformations, has been associated with a high mortality rate since its identification in 2008. This report presents the case of a remarkable patient who recently celebrated her fifth birthday, marking her as the oldest documented individual with this syndrome to date. Highlighting the significance of comprehensive evaluations for concurrent malformations, the report discusses potential treatment modalities and challenges inherent in managing patients with this intricate syndrome. A comprehensive review of previously published cases is provided, comparing surgical interventions, causes of death, and age at the time of demise. This report underscores the importance of ongoing research and collaborative efforts to optimize outcomes for individuals afflicted with serpentine-like syndrome.

## 1 Case description

A 28-year-old primigravid mother presented to our institution with a prenatal diagnosis of a liver-down diaphragmatic hernia and polyhydramnios. The observed/expected lung-to-head ratio was 64% at 32 weeks gestational age. Because of the good prognosis neither magnetic resonance imaging (MRI) nor Fetal endoscopic tracheal occlusion (FETO) were performed. At 37 weeks’ gestation, a cesarean section was performed for premature rupture of the membranes, and a female infant was born with a birth weight of 2,490 g (5th percentile), length of 49 cm (40th percentile), and head circumference of 34 cm (50th percentile). The APGAR-Scores were 7/9/9. On first inspection, the abdomen appeared sunken-in and the head seemed to be placed directly on the shoulders with a short neck. No other external malformations were observed. Primary intubation and High Frequency Oscillatory Ventilation (HFOV) were initiated. The patient experienced arterial hypotension unresponsive to catecholamines and hydrocortisone, with improvement only after a therapeutic trial of dexamethasone. The brain ultrasound (US) was without pathological findings. The echocardiography showed a small ventricular septum defect without hemodynamic relevance. The abdominal US showed a central, large liver-down-diaphragmatic defect with intrathoracic stomach and bowel. Both kidneys and adrenal glands appeared normal in size, configuration, and position. Liver and gallbladder were also normal. The pancreas could not be identified and was suspected to be intrathoracic. The suspicion of asplenia was confirmed by the sonographic absence of the spleen and the presence of Howell-Jolly bodies. The x-Rays of chest and abdomen showed pulmonary hypotransparency, an atypically configured heart, and the gastric tube ending in the right hemithorax suggesting intrathoracic location of the stomach. No intestinal gas was observed radiographically on day of birth.

**Figure 1:**
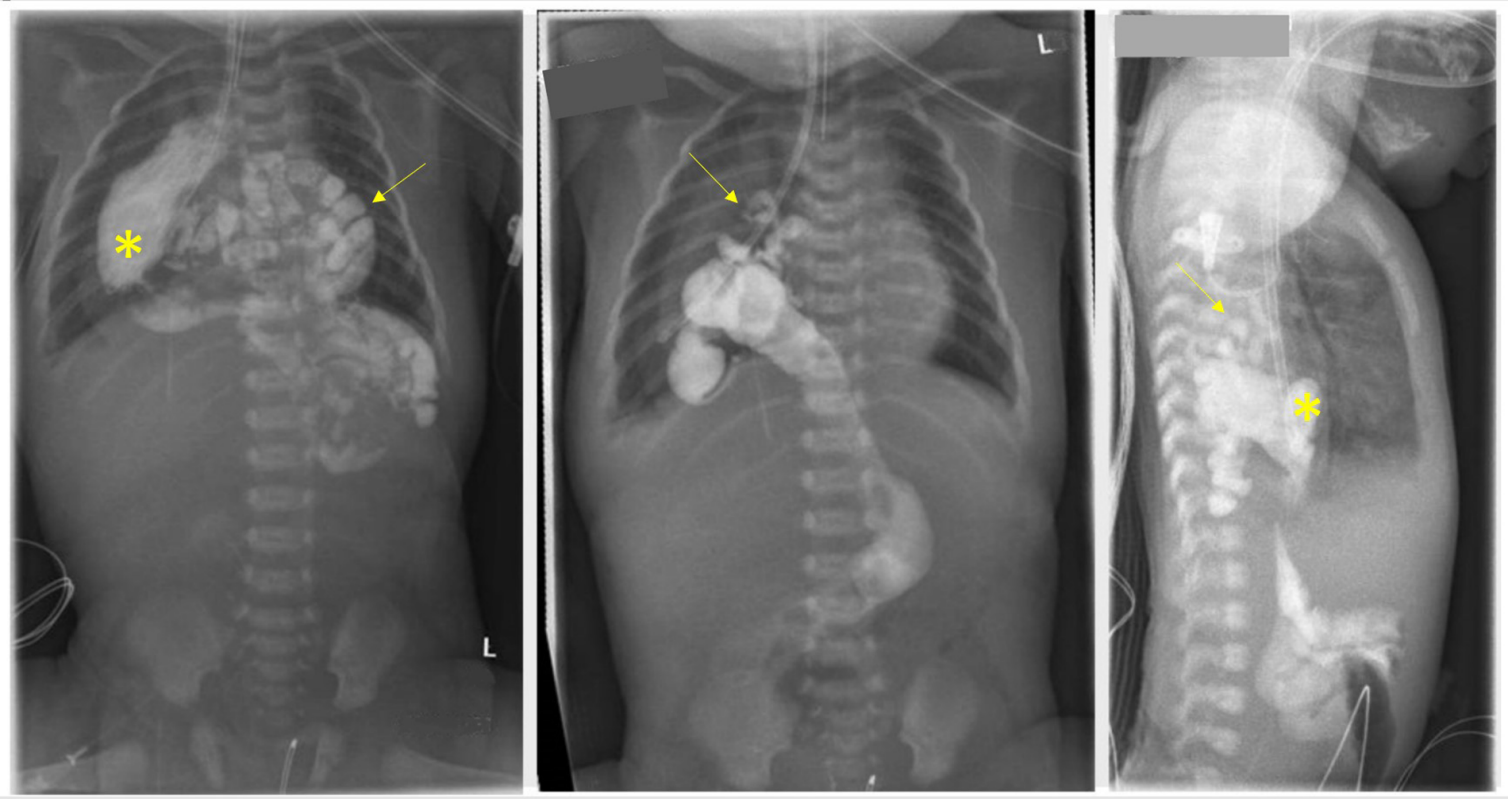
The upper gastrointestinal contrast study conducted on the third day of life confirmed the intrathoracic positioning of the stomach (asterisk) within the right hemithorax. Additionally, segments of the jejunum, ileum, proximal colon, and appendix (arrow) were also found to be located intrathoracically.

Therefore, after stabilization of the patient an upper gastrointestinal contrast study (upper GI study) was performed at her 3rd day of life (see Figure 1) to rule out gastrointestinal atresia. The study confirmed the US findings and the almost completely intrathoracic bowel.

**Figure 2:**
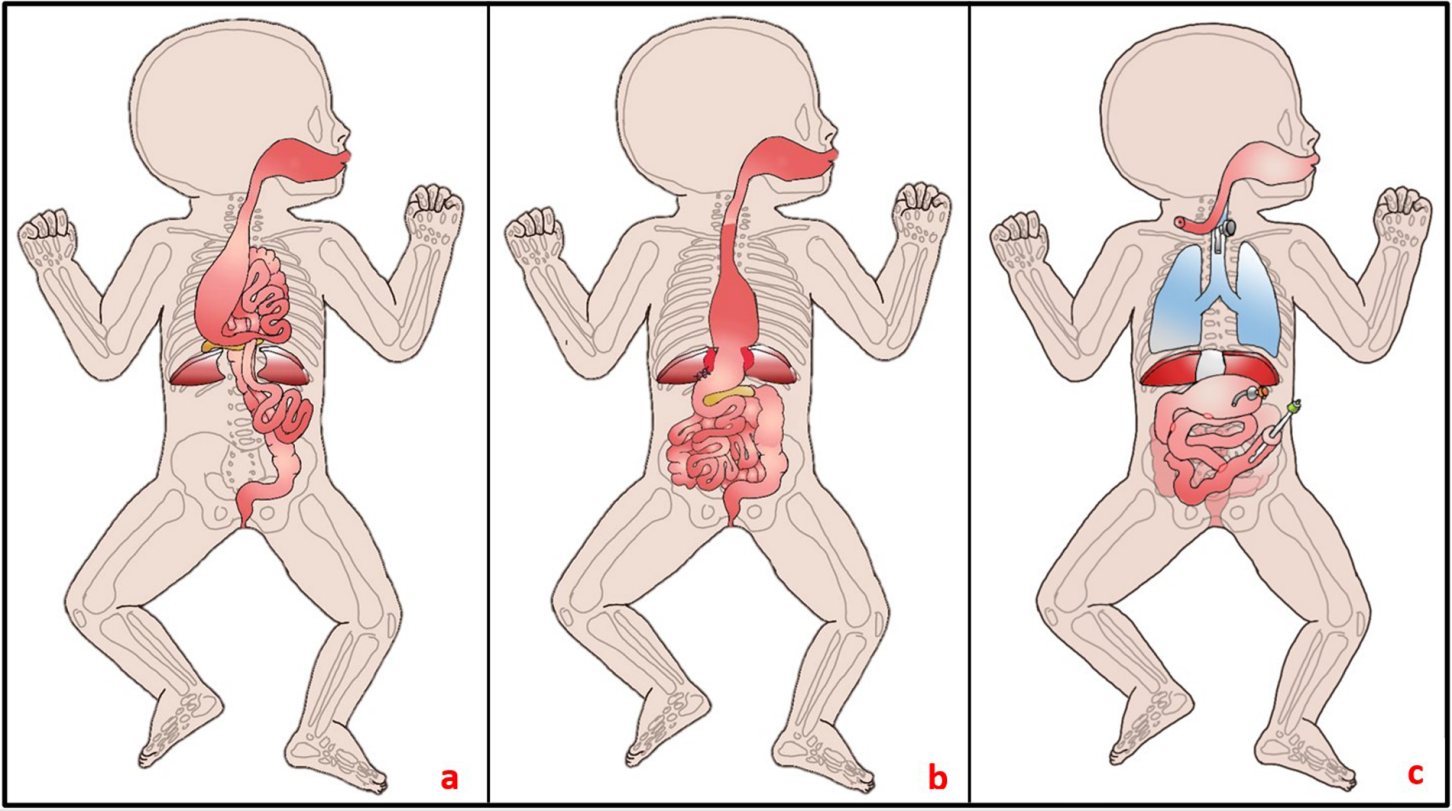
**(a)** Intraoperative findings on the 4th day of life: A substantial portion of the bowel protruded into the thorax through the central diaphragmatic defect. The stomach appeared small and was attached to a brachyoesophagus, while the pancreas was situated intrathoracically. No spleen was identifiable. **(b)** During the initial surgery, efforts were made to relocate the bowel into the abdomen; however, due to a short esophagus, only small portions of the stomach could be repositioned, resulting in fixation at the diaphragm. **(c)** Current status at 13 months of age: Notable features include a tracheostomy, collar esophageal fistula, closed diaphragmatic defect, gastrostomy, gastrojejunostomy, duodenojejunostomy, and jejunostomy, the latter performed as a Roux-Y-anastomosis.

**Figure 3:**
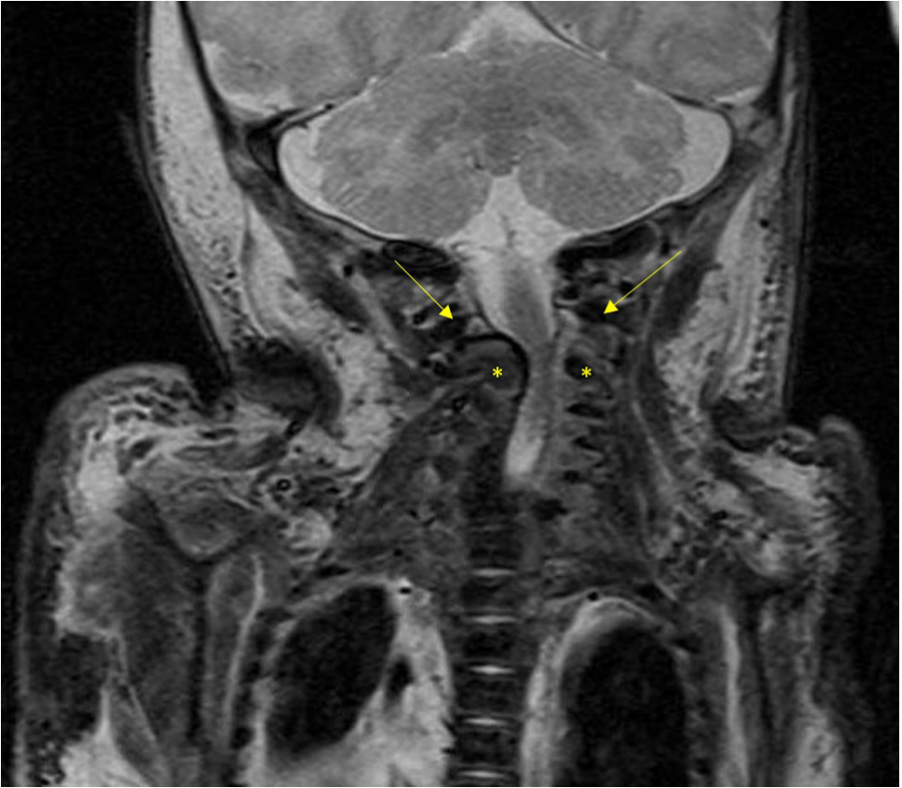
The MRI image illustrates compression of the spinal cord by the atlas (arrow) and axis (asterisk), resulting in a significant narrowing of the spinal canal to 5 mm.

On the 4th day of life, we performed a midline laparotomy. Intraoperative discoveries were mostly those seen in US and upper GI study except for that the stomach was not only small but also attached to a very short esophagus (see Figure 2). The pancreas was also situated in the thorax. The herniated bowel placed in the abdomen, an incidental appendectomy was performed, and the diaphragmatic defect was closed. The esophagus was so short that only one-third of the stomach could be placed in the abdomen. To prevent recurrent herniation into the thorax, the stomach was attached to the diaphragm. Postoperatively, the patient was transferred back to the pediatric intensive care unit (PICU), and the multidisciplinary team discussed the case. Intraoperative atypical findings and a thorough review of the literature led us to believe that the patient could have a serpentine-like-syndrome (SLS), a rare condition with only few reported cases. Since the syndrome is usually associated with cervical vertebrae anomalies and frequently the need of neurosurgical intervention, we performed an MRI. A pronounced rachischisis of the cervical spine was found, in addition to a compression of the spinal cord by the atlas and axis with an absolute narrowing of the spinal canal to 5 mm (see Figure 3). Neurosurgical treatment included laminectomy of the second cervical body and cervical spinal cord decompression on day 17 of life. Retrospectively, cardiovascular instability immediately after birth, which stabilized only after administration of dexamethasone, correlated as a sign of spinal shock at birth.

A whole-genome sequencing was performed which did not reveal any abnormalities.

At 1 month of age, she was extubated and enteral feeding was started at the same time. Because of increasing bilious gastric reflux and signs of dysmotility as well as delayed gastric emptying, we performed a duodenojejunostomy shortly after. The symptoms persisted, and she could not be adequately fed so we performed a jejunostomy as a Roux-Y-Anastomosis in the stoma. It was then possible to feed her this way. However, oral intake remained low, with much reflux and discomfort for the patient after oral feeding. Another contrast study at 4.5 months of age showed a duodenal obstruction at the level of the diaphragm. Therefore, a gastrojejunostomy was performed. Intraoperatively, a reopening of the diaphragmatic defect was noted and closed with a Gore-Tex patch. When a feeding routine was established and tolerance of enteral nutrition with adequate weight gain was assured, she was discharged.

At the age of 10 months, she presented due to severe discomfort during and shortly after oral feeding as well as pulmonary problems. Further diagnostics confirmed atelectasis, perhaps because of lung tissue compression by the stomach during feeding or micro-aspirations. A Collis gastroplasty was planned, but due to the small stomach, it was decided intraoperatively to dissociate the esophagus and stomach and close the diaphragm defect completely. To administer medication and possibly food a gastrostomy was formed. Afterwards feeding was unproblematic and the patient gained weight sufficiently. A collar fistula was established at 11 months of age due to excessive salivation and recurrent respiratory infections. The pulmonary situation remained problematic; it was impossible to wean the patient of ventilation. Consequently, our PICU and Otorhinolaryngology (ENT) specialists recommended a permanent tracheostomy, which was performed at 13 months.

## 2 Case report timeline


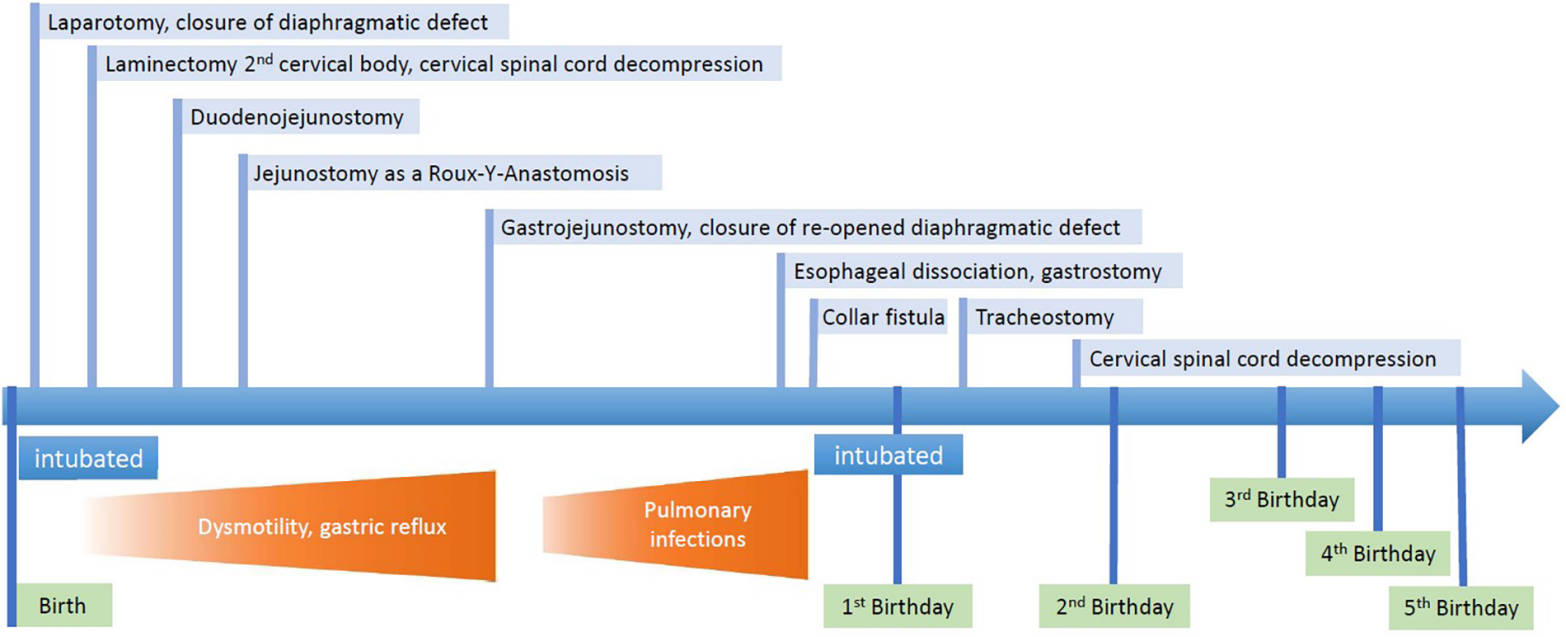


## 3 Follow-up and outcome

The patient was discharged home after the parents were trained in handling all the equipment and a pediatric intensive-care service was established. Since then, the girl was regularly presented to our hospital for check-ups and tracheoscopies. She is connected to a social pediatric center close to her home and is making great developmental steps. This year she joined an inclusion kindergarten. She is able to stand, speaks in more-word-sentences, and draws using a tripod pencil grasp.

During the infection season she is a frequent guest on our PICU where she sometimes needs improved ventilation. When she is at home she is ventilated at night for a few hours. Feeding is continuously administered 17 h per day via her jejunostomy. She receives penicillin for asplenia-prophylaxis. At 9 months she crossed the 3rd weight percentile and has since been stable around the 25th percentile. A care service provides 24-h-support to the family.

At this point the family and we are happy to maintain a stable situation. Prospects in the future are weaning of the ventilation and removal of the tracheostomy as well as maybe a restoration of the oral-enteral continuity, perhaps with a jejunal interposition. For such a complex surgical procedure with an associated long hospital stay we plan to wait for further stabilization to not throw her back in her development too much. Even though SLS is described as a disease mostly fatal in the first year of life the patient has recently celebrated her 5th birthday.

## 4 Discussion

The congenital combination of brachyoesophagus, intrathoracic stomach, spinal abnormalities and cervical spine deformity was named SLS due to snakes’ similar anatomy. A snake's stomach is located in their thorax and the cervical vertebrae are not completely united dorsally. Since the first description of SLS in 2008 by Katz et al. (1) only few cases have been described in the English literature. The last overview by Song et al. (2) included 10 patients. An additional case described by Thakker and Donnai (3) showing similar but also many additional malformations was included in the following analysis. Considering these patients, two cases which appear to describe SLS without naming it as such and our case, only 14 reports of patients with SLS are published so far (see Table 1).

**Table 1:** List of cases with SLS and their respective outcomes.

Case number	Oldest reported age	Outcome	Cause of death	Therapy/time after birth
1. Leung et al. (4)	4 days	Death after operation	Withdrawal of intensive care due to non-correctable gastrointestinal malformations	4 days: emergency laparotomy
2. Katz et al. (1)	Three years	Alive after operation		13 days: laparoscopy and jejunostomy tube
				5 weeks: dissection of the intrathoracic stomach, gastrostomy
				18 months: collis gastroplasty
3. Katz et al. (1)	Newborn	Death without operation	Withdrawal of life support due to inoperable and cardiac abnormalities	
4. Deprez et al. (5)	2 years	Death after operation	Acute purulent tracheobronchitis causing complete obstruction of the prosthesis	1 week: explorative laparotomy, laterolateral duodenojejunostomy, cholecystojejunostomy
				6 months: reduction of the stomach by tubulization (right thoracotomy)
				18 months: tracheal prosthesis
5. Nakamura et al. (6)	6 months	Death after operation	Acute cardiopulmonary insufficiency on the 10th postoperative day	15 days: tracheostomy
				2 years: jejunostomy
				6 months: gastric tubularization, reduction of herniated organs, hiatus plasty
6. Dargan et al. (7)	51 days	Death after operation	Respiratory complications under palliative care	3 days: laparotomy and right thoracotomy, proximal small bowel stoma
7. Dorum et al. (8)	Newborn	Alive after operation		Unknown: jejunostomy
8. Beleza-Meireles et al. (9)	12 days	Death without operation	Unknown	
9. Mimura et al. (10)	20-week gestation	Died prenatally	Termination of pregnancy	
10. Song et al. (2)	23-week gestation	Died prenatally	Termination of pregnancy	
11. Thakker and Donnai (3)	25-weeks gestation	Died prenatally	Termination of pregnancy	
12. Xia et al. (11) not described as SLS	12 months	Alive after operation		3 days: gastropexy, gastric folding antireflux procedure and pyloroplasty
13. Winckworth et al. (12) not described as SLS	4 months	Death after operation	Withdrawal of life support due to sepsis	2 days: laparotomy, feeding jejunostomy
				6 weeks: thoracotomy, pyloromyotomy, partial gastrectomy
Our case	5 years			See above

Please note that the “oldest reported age” denotes the age specified by the authors in the referenced publication, which may not always correspond to the exact age at the time of death.

In addition, the extremely rare association of brachyoesophagus with intrathoracic stomach has been described outside the context of SLS. In 2009, a case showed these defects associated with an intrathoracic spleen with an intact diaphragm and Iniencephaly, a rare cervical neural tube defect (12). After multiple surgical procedures, the patient died of sepsis at 4 months of age. More recently, a case report (11) described the association without additional malformations, but defects in thoracic vertebral segmentation are evident on published radiologic images. Surgical treatment consisted of left diaphragmatic repair, gastropexy, gastric fold antireflux procedure, and longitudinal incision and transverse suture for pyloroplasty. The authors report that the patient reached the first year of life with severe gastroesophageal reflux as the only complication described. The extreme rarity of the syndrome, its recent description, and the wide range of penetrance make it difficult to recognize.

In cases the pregnancy was not terminated after the prenatal diagnosis of the malformations the syndrome is often fatal shortly after birth. The reasons for passing of the child differ from case to case (see Table 1). A statistical analysis of the causes of death is not possible due to the low number patients but in the reports published so far pulmonary problems are the most common fatal complication in children with SLS.

To date, there are no reports on the quality of life of patients who have survived the neonatal period, which makes counseling and therapeutic adjustment difficult. Similarly, the varying degrees of severity of associated malformations require individualized counseling and treatment.

SLS is a rare syndrome with a high expenditure and the need of interdisciplinary teams including pediatric surgeons, neonatologists and PICU professionals, pediatric neurosurgeons, pediatric ENT specialists, nutritionists, physiotherapists and many more. Discussing patients like these with colleagues from other clinics helps finding the most accurate therapy. Since the syndrome is extremely rare, a thorough search for concomitant malformations is indispensable.

Our example shows how other reports can draw our attention to malformations which might have been overlooked otherwise. In our case the diagnosis of a specific syndrome, even though the mortality in other case reports is high, helped the parents deal with the diagnosis and having further information about the disease helped them to arrange with the long-term hospitalizations of their daughter.

As well as our patient other patients suffered from gastric outlet obstruction and prolonged gastric emptying as well as reflux. Feeding via jejunostomy was in some case a sufficient therapy to establish enteral nutrition. The presence of skilled parenteral nutritionists was crucial, particularly in instances where enteral feedings were inadequate.

Pulmonary complications like recurrent infections, atelectasis and impossible weaning from ventilation may lead to tracheostomy. No reports of decannulation and closure of the tracheostomy in SLS patients are available. The possible need for long-term-ventilation and the associated risks of a tracheostomy (5) must be taken into consideration when planning the therapy of these patients. Given the significant impact of pulmonary infections, it is imperative to ensure their prompt diagnosis and adequate treatment, particularly in light of the high mortality associated with them.

## Data Availability

The original contributions presented in the study are included in the article/Supplementary Material, further inquiries can be directed to the corresponding author.
